# Medical Report of the Neilgherry Mountains

**Published:** 1823-03

**Authors:** R. Orton

**Affiliations:** Assistant Surgeon 34th Regt.


### Art. V.—*Medical Report of the Neilgherry Mountains.* By R. Orton, Esq. Assistant Surgeon 34th Regt.

This tract of country is about twenty miles in breadth from north to south, and fifty in length from east to west. It is bounded on the north by Mysore, on the east and south by Coimbatoor, and on the west by Malabar. Its forms a perfect *table-land*, its edges being well defined, and generally rising abruptly to the height of several thousand feet above the plain, and the lowest valleys on its surface being much higher than the neighbouring country. It is completely insulated, except, perhaps, at its western extremity, where the line of western ghants appears to be continued across it; but it is said to be completely separated on both sides from these also. The general elevation cf the eastern half of the range, and a large tract of the north-west part, appears to be between five and six thousand feet above the sea; but, on proceeding to the interior and western part, either from the east or north edge, we arrive at another abrupt ascent of fifteen hundred or two thousand feet, which may be termed the second edge of the table-land. On ascending this, we arrive at a very extensive and pretty level tract, forming the top of the table-land, which is termed the Sodiernaad. Beyond this, at a great distance to the westward, are seen a great range of mountains still higher, which appear to be situated about the western extremity of the Neilgherry, and to be a part of the great line of the western ghants.

The strata of the Neilgherry are very similar to those of the Malabar coast in general; consisting chiefly of a great layer of laterite, in various states, over the basis of granite. Above this, again, are frequently found strata of black earth, resembling the cotton-soil of the low country, and vegetable mould; but these are in very many places wanting, the laterite forming the surface, or nearly so; and, as it is very gravelly and unfit for vegetation, it gives the hills rather a bare appearance.

The surface of the whole of the less elevated part of the country consists of a continued series of steep hills and mountains, without any level ground. It is almost entirely covered with short, thin brushwood and grass, except where cultivated; and has no swamps, and very few woods, except at the extreme edges of the table-land. The Sodiernaad has, however, a different appearance: it consists chiefly of swells of moderate height, quite free from brushwood, but interspersed with numerous swamps, and patches of high and thick jungle, where much vegetable putrefaction is continually going on. It is entirely uncultivated; but its soil appears very rich, as a thick layer of brown vegetable mould appears almost every where at the surface, producing thick short grass.

The inhabitants of the Neilgherry consist of three different castes of Hindoos,—Bergers, Cotters, and Joders; all living in separate villages. The Cotters are a black and miserable looking race of the lowest cast, and much resembling the Chucklers below. The Bergers, who compose the principal part of the population, are a better looking race, and bear no appearance of the effects of an unhealthy climate: their complexion is rather lighter than that of the Ryots below; but I cannot say that they exhibit either the fairness or muscularity one would expect to find in the inhabitants of a temperate climate. Many of them have, however, much of the European contour of features, and particularly the Grecian nose.

The Joders are a very remarkable set of people. Their stature is equal to that of Europeans, and they are nearly as muscular; so that they differ very widely in these respects from other natives of India. Their features are so regular, that, in a whole village, scarcely a man could be found who might not be termed handsome. Their dispositions, too, appear to be very different from those of other natives: they have a continued and excessive flow of spirits; and two of them became so much attached to Mr. Stoddart and myself, that they actually shed tears on our departure. They inhabit only the Jodiernaad. They live solely by attending buffaloes, and never cultivate the ground, migrating frequently from place to place on account of the grass. By their own account, they are a very healthy race.

The weather in June, when I ascended the mountains, was fine for about one-third of the month; the rest very rainy. In July, several light showers fell almost every day, and there was little sun-shine; but no great quantity of rain fell. August was fine almost throughout. September, October, and the beginning of November, were very rainy, cloudy, and foggy; the intervals of fine weather being very few, and not more than a day or two at a time. The falls were often very heavy and continued. In October, the prevailing wind changed from west to east. I was informed that the months of November and December are also usually very rainy; the N.E. monsoon prevailing in at least an equal degree with S.W.; but, in the latter part of December, the atmosphere becomes clear and very cold, and continues so all January, February, and March; and all accounts speak of those months as being most delightful and healthy. April and May are rather showery, but perfectly temperate. The S.W. monsoon sets in the beginning of June. It may be said therefore, in short, that the first three months of the year are dry, clear, and very cold; the two next chiefly fine and cool, but rather showery; and the remaining seven, for the most part, rainy, foggy, and cloudy, but with considerable intervals of fine weather.

The changes in the weather are often extremely sudden: the sky, from being perfectly serene and bright, becoming all at once overcast by immense clouds, which, after a little while, disappear again as suddenly as they came on; and this, perhaps, several times in the day. The weather, when fine, certainly is delightful. There is something highly exhilarating in breathing so rarefied an air, when it is clear.

The sky, when clear, exhibits a deep blue colour, such as we never see below; and, under the same circumstances, distant objects appear uncommonly distinct and bright. It is also a strong fact in favour of the climate, that iron is slow in acquiring rust, even in rainy weather; at least, in the S.W. monsoon. Thunder and lightning seemed to be of rather rare occurrence, as we observed them only three or four times in a slight degree at the breaking-up of fine weather. It is a very prevalent opinion among the natives of the low country about the Neilgherry that its climate is extremely unhealthy, and they have, consequently, the greatest dread of ascending it. I find also that Dr. Buchanan mentions the existence of a similar report, at the time of his visiting that neighbourhood, (about 1800.) It is said that this report is owing to the false accounts of the renters of the hills, whose interest it is to keep Europeans in ignorance of them, and to the disagreeable impressions which natives receive from entering suddenly into so cold a climate in their half-naked state. I fear, however, that it is not entirely, though in a great measure, contradicted by late experience. It appears sufficiently evident that some disposition to produce fever does exist in this climate: the principal question regarding it is the degree in which this cause prevails. From all that I have seen and heard on this subject, I should conclude that it certainly does not exist there in a greater degree than in the rest of the province of Coimbatoor, or even in the less unhealthy parts of Mysore; and the low temperature of the climate must, and does, prove highly beneficial to many persons labouring under the diseases produced by a high one. But I will put the reader in possession of all the facts which have led me to these inferences.

I have incidentally met with a few cases of fever among the Berger and Cotter villagers, and their deaths appear to be rather too numerous. Six persons died in the village of Dimhutty, consisting of about thirty families, during the five months which I remained on the hills. The Cotter village, in the same neighbourhood, suffered at least as much. But the food and clothing of these poor people seems to be so inadequate to the wants of nature, that they could scarcely be expected to be quite healthy in any climate. In the Joder village of Wuttacamund, I was informed that no death had happened for three years. A large party of pioneers and coolies, employed in making a road up the Neilgherry, continued healthy, excepting rather frequent, but slight, attacks of fever during the year they were employed on the table-land; but have suffered extremely since they began to work near the bottom of it. Three-fourths of the servants and followers of four officers, who went up in June last, (of whom I was one,) were very shortly attacked with fever, but all recovered; and, after two or three months, those who remained no longer suffered returns of it. A remarkable difference was found in the case of the very numerous followers of two officers, who went up in July and August; for, though the circumstances were in other respects similar, not more than two or three of them suffered attacks during three months they remained.

From these facts, and observations on my own case, it appeared very strongly that the first month of my residence on the hills (June,) was less healthy than the remaining four. It is to be remarked, that the S.W. monsoon set-in in June; and it is probable that the climate became more healthy in consequence of the first heavy rains, as is generally observed to be the case in hilly and jungly countries,—particularly at Courtalum, the climate of which probably bears a great analogy to that of the Neilgherry.

It appeared to me, that many of the attacks of fever which occurred among our followers were owing to exposure to cold and damp, the predisposing or principal cause having been contracted elsewhere; and that, therefore, the climate, in such cases, was no more to be blamed than that of Bangalore is for the very numerous attacks of fever which occur there in persons arriving from Seringapatam. This opinion was strengthened by the circumstance of most of these attacks happening almost immediately after their arrival on the Neilgherry. The operation of the great cause of endemic fever appears to be much more gradual; for a corps generally remains healthy for several months after arriving at Seringapatam. Again, we found that these attacks in our followers almost entirely ceased to recur after we had been some time on the hills; whilst, at places where fever is endemic to a great extent, as at Seringapatam, the reverse is the case. The sudden transition, too, from a very high to a low temperature, which is generally experienced on ascending these mountains, doubtless is a great cause of the attacks of fever which occur. Thus, when I ascended them, it was about the hottest season in the year; the thermometer, in the low country of Coimbatoor, getting as high as 98, and not falling lower than 82; but, after a march of ten miles to Jackanary, we found the temperature no higher than 72 in the afternoon, and as low as 60 at night. It is no wonder, therefore, that many attacks of fever were produced in persons who had no defence against the cold but a *cumly* or two,—who all slept on the ground,—and many of them in an open shed, with the wind blowing on them.

These circumstances evidently suggest the necessity for persons, visiting the Neilgherry, carefully guarding against exposure to cold and damp. I believe it will rarely be found that the attacks are not to be traced to some cause of that kind.

The exciting cause of my first attack appeared to arise from walking for some distance in a heavy dew, among long grass and shrubs. Another officer was attacked with fever on the day after his having got wet in a shower, and neglected to change his clothes. It is also necessary for the invalid to avoid much exposure to the sun; for, though it is seldom so powerful as to be felt inconvenient, it always burns the face almost as much as in the low country, and I know, from experience, that it is very injurious to health and productive of fever.

[To be continued.]

---

Art. VI.—*Case of Acute Rheumatism translated to the Heart.* By Andrew Armstrong, Esq. Assistant Surgeon, Grenadier Regiment of Foot Guards.

